# Supplementary material for: Approximate Inference for Time-Varying Interactions and Macroscopic Dynamics of Neural Populations
Source: PLoS Comput Biol. 2017 Jan 17;13(1):e1005309. doi: 10.1371/journal.pcbi.1005309 (PMC5283755; doi:10.1371/journal.pcbi.1005309)
Supplement: S1 Text — (PDF) [file pcbi.1005309.s001.pdf]

Approximate Inference for Time-varying Interactions and Macroscopic Dynamics of Neural Populations

Christian Donner[1, 2, 3], Klaus Obermayer[1, 2], Hideaki Shimazaki[4]

1 Bernstein Center for Computational Neuroscience, Berlin, Germany
2 Neural Information Processing Group, Department of Electrical Engineering and Computer Science, Technische Universität Berlin, Berlin, Germany
3 Group for Methods of Artificial Intelligence, Department of Electrical Engineering and Computer Science, Technische Universität Berlin, Berlin, Germany
4 RIKEN Brain Science Institute, Wako-shi, Saitama, Japan

* shimazaki@brain.riken.jp

S1 Text. Bethe approximation.

The Bethe approximation, belief propagation (BP), and concave convex procedure (CCCP) are well explained by [1–3]. However, for the sake of consistency the methods are summarized here once more. First the Bethe approximation in general will be discussed and subsequently the two algorithms to find its solution.

The Bethe approximation is a variational approach. One assumes that the joint distribution of the Markov network can be written in terms of its individual and pairwise marginals

$$q(\mathbf{x}) = \frac{\prod_{i,j>i} q(x_i, x_j)}{\prod_i q(x_i)^{N_i - 1}}, \tag{1}$$

where N_i is the number of neighbors of neuron i. Eq 1 ignores any cycles in the network and would be exact for a tree. The aim is to find the distribution $q(\mathbf{x})$ that is closest to our actual one $p(\mathbf{x}) = \exp(\boldsymbol{\theta}'\mathbf{F}(\mathbf{x}) - \psi)$, i.e., the one that minimizes the Kullback-Leibler (KL) divergence

$$D_{\mathrm{KL}}(q\|p) = \sum_{\mathbf{x}} q(\mathbf{x}) \log \frac{q(\mathbf{x})}{p(\mathbf{x})} = \phi(q) - \boldsymbol{\theta}' \left\langle \mathbf{F}(\mathbf{x}) \right\rangle_q + \psi, \tag{2}$$

where $\langle . \rangle_q$ is the expectation over $q(\mathbf{x})$ and $\phi(q)$ its negative entropy. The Bethe approximation of the log partition function is given by

$$\psi \approx \psi_{\mathrm{Bethe}} = \psi - D_{\mathrm{KL}}(q\|p) = -\phi(q) + \boldsymbol{\theta}'\langle \mathbf{F}(\mathbf{x}) \rangle_q. \tag{3}$$

Eq 3 shows the nature of the approximation error. As long as the class of distribution $q(\mathbf{x})$ contains distributions close to the actual $p(\mathbf{x})$ the error will be small, because the KL divergence will be small. Furthermore, we see that ψ_{Bethe} will underestimate ψ systematically because $D_{\mathrm{KL}} \geq 0$. Eq 2 provides an objective function that needs to be minimized w.r.t. $q(\mathbf{x})$. Realizing that ψ does not depend on $q(\mathbf{x})$, the problem is equivalent to maximizing Eq 3. Furthermore, $q(x_i)$ and $q(x_i, x_j)$ must fulfill following constraints:

$$q(x_i) = \sum_{x_j} q(x_i, x_j) \text{ for } i = 1, \ldots, N, j \neq i \tag{4}$$

Normalization constraints for the marginals are ignored for the moment. The problem can be written as a Lagrangian

$$\mathcal{L}(q) = \psi_{\text{Bethe}} + \sum_{i \neq j} \sum_{x_i} \lambda_j(x_i) \left(\sum_{x_j} q(x_i, x_j) - q(x_i) \right). \tag{5}$$

By setting the derivative w.r.t. $q(x_i)$ and $q(x_i, x_j)$ to 0, the marginals can be expressed in terms of the Lagrangian multipliers

$$\begin{aligned} q(x_i, x_j) &\propto \exp\left(\theta_i x_i + \theta_j x_j + \theta_{ij} x_i x_j + \lambda_j(x_i) + \lambda_i(x_j)\right), \\ q(x_i) &\propto \exp\left(\theta_i x_i + \frac{\sum_{i \neq j} \lambda_j(x_i)}{N_i - 1}\right). \end{aligned} \tag{6}$$

This constitutes the Bethe approximation and it remains to find the marginals $q(x_i)$ and $q(x_i, x_j)$. In the following subsections two procedures are described, that diverge from this point.

Belief propagation The BP starts from Eq 6, but writes the Lagrangian multipliers in terms of messages as

$$\lambda_j(x_i) = \log \prod_{k \in N(i) \setminus j} m_k(x_i). \tag{7}$$

$N(i) \setminus j$ are the set of neighbors of i without j, and $m_k(x_i)$ is the *message* sent from node k to i. Substituting this into Eq 6 yields

$$\begin{aligned} q(x_i, x_j) &\propto \exp\left(\theta_i x_i + \theta_j x_j + \theta_{ij} x_i x_j\right) \prod_{k \in N(i) \setminus j} m_k(x_i) \prod_{k \in N(j) \setminus i} m_k(x_j), \\ q(x_i) &\propto \exp\left(\theta_i x_i\right) \prod_{k \in N(i)} m_k(x_i). \end{aligned} \tag{8}$$

By substituting these marginals into Eq 4 a set of self-consistent equations for the messages can be obtained

$$m_j(x_i) = \sum_{x_j} \exp\left(\theta_j x_j + \theta_{ij} x_i x_j\right) \prod_{k \in N(j) \setminus i} m_k(x_j). \tag{9}$$

The BP algorithm initializes the messages and solves Eq 9 iteratively until the algorithm converges. Having obtained the messages, the marginals can be computed by Eq 8 and they just need to be normalized in the end.

Concave convex procedure While the BP algorithm takes care of the normalization constraints only in the end and hence does not sometimes converge, the CCCP [3] is more strictly about them, which guarantees convergence at the cost of computation time.

The starting point is the Lagrangian function depicted in Eq 5. Instead of maximizing ψ_{Bethe} with the constraints, here we follow [3] that minimizes the Gibbs free energy, which is $-\psi_{\text{Bethe}}$. Furthermore, the normalization constraint

$$\sum_{x_i, x_j} q(x_i, x_j) = 1, \tag{10}$$

is added, resulting in the Lagrangian

$$\mathcal{L}_{\text{CCCP}}(q) = -\psi_{\text{Bethe}} + \sum_{i\neq j}\sum_{x_i}\lambda_j(x_i)\left(\sum_{x_j} q(x_i,x_j) + q(x_i)\right) + \sum_{i\neq j}\gamma_{ij}\left(\sum_{x_i,x_j} q(x_i,x_j) - 1\right). \tag{11}$$

The basic principle of the CCCP is to realize that $-\psi_{\text{Bethe}}$ can be decomposed into a convex and a concave part

$$-\psi_{\text{Bethe}} = \underbrace{\sum_{i\neq j}\sum_{x_i,x_j} q(x_i,x_j)\log\frac{q(x_i,x_j)}{\exp(\theta_i x_i + \theta_j x_j + \theta_{ij}x_i x_j)} + \sum_i\sum_{x_i} q(x_i)\log\frac{q(x_i)}{\exp(\theta_i x_i)}}_{F_{\text{convex}}} \underbrace{- \sum_i N_i \sum_{x_i} q(x_i)\log\frac{q(x_i)}{\exp(\theta_i x_i)}}_{F_{\text{concave}}}. \tag{12}$$

Calculating the derivative w.r.t. the marginals yields the following iterative update rule for q

$$\begin{aligned}\frac{\partial}{\partial q(x_i,x_j)} F_{\text{convex}}(q^{t+1}) &= -\frac{\partial}{\partial q(x_i,x_j)} F_{\text{concave}}(q^t) - \lambda_i(x_j) - \lambda_j(x_i) - \gamma_{ij},\\ \frac{\partial}{\partial q(x_i)} F_{\text{convex}}(q^{t+1}) &= -\frac{\partial}{\partial q(x_i)} F_{\text{concave}}(q^t) + \sum_k \lambda_k(x_i).\end{aligned} \tag{13}$$

Note, that here t is an integer describing the iterations of the algorithm and not the time-dependence of the model. By updating the marginals with Eq 13, $-\psi_{\text{Bethe}}$ monotonically decreases (see Theorem 2 in [3]). Writing the update explicitly for the marginals, we get

$$\begin{aligned} q^{t+1}(x_i,x_j) &= \exp\left(\theta_i x_i + \theta_j x_j + \theta_{ij}x_i x_j - \lambda_i(x_j) - \lambda_j(x_i) - \gamma_{ij}\right),\\ q^{t+1}(x_i) &= \left(\frac{q^t(x_i)}{\exp(\theta_i x_i)}\right)^{N_i} \exp\left(\theta_i x_i + N_i + \sum_j \lambda_j(x_i) - 1\right).\end{aligned} \tag{14}$$

Assume we have a set of Lagrangian multipliers such that the constraints in Eq 4 and 10 are satisfied. Then $-\psi_{\text{Bethe}}$ can be decreased by updating the marginals with Eq 14. However, by doing so the constraints will be violated and one has to update the Lagrangian multipliers. By substituting Eq 14 into the constraints (Eq 4 and 10), one gets self-consistent equations for the multipliers that write as

$$\begin{aligned} \exp(\gamma_{ij}) &= \sum_{x_i,x_j} \exp\left(\theta_i x_i + \theta_j x_j + \theta_{ij}x_i x_j - \lambda_j(x_i) - \lambda_i(x_j) - 1\right),\\ \exp\left(2\lambda_j(x_i)\right) &= \frac{\sum_{x_j}\exp(\theta_j x_j + \theta_{ij}x_i x_j - \lambda_i(x_j) - \gamma_{ij})}{\left(\frac{q^t(x_i)}{exp(\theta_i x_i)}\right)^{N_i}\exp\left(N_i + \sum_{k\neq\{i,j\}}\lambda_k(x_i)\right)}.\end{aligned} \tag{15}$$

The multipliers are updated sequentially until the constraints for the marginals are again satisfied.

The CCCP always updates first the marginals. For each update the Lagrangian multipliers have to be updated until the constraints are fulfilled again. This alternating procedure is done until the Bethe free energy converges.

References

1. Yedidia J. An idiosyncratic journey beyond mean field theory. Advanced mean field methods: Theory and practice. 2001; p. 21–36.
2. Yedidia JS, Freeman WT, Weiss Y. Understanding belief propagation and its generalizations. Exploring Artificial Intelligence in the New Millennium. 2003;8:236–239.
3. Yuille AL. CCCP algorithms to minimize the Bethe and Kikuchi free energies: Convergent alternatives to belief propagation. Neural Computation. 2002;14(7):1691–1722.
