# Supplementary material for: Approximate Inference for Time-Varying Interactions and Macroscopic Dynamics of Neural Populations
Source: PLoS Comput Biol. 2017 Jan 17;13(1):e1005309. doi: 10.1371/journal.pcbi.1005309 (PMC5283755; doi:10.1371/journal.pcbi.1005309)
Supplement: S2 Text — (PDF) [file pcbi.1005309.s002.pdf]

Approximate Inference for Time-varying Interactions and Macroscopic Dynamics of Neural Populations

Christian Donner[1, 2, 3], Klaus Obermayer[1, 2], Hideaki Shimazaki[4]

1 Bernstein Center for Computational Neuroscience, Berlin, Germany
2 Neural Information Processing Group, Department of Electrical Engineering and Computer Science, Technische Universität Berlin, Berlin, Germany
3 Group for Methods of Artificial Intelligence, Department of Electrical Engineering and Computer Science, Technische Universität Berlin, Berlin, Germany
4 RIKEN Brain Science Institute, Wako-shi, Saitama, Japan

* shimazaki@brain.riken.jp

S2 Text. TAP approximation.

The *Thouless-Anderson-Palmer* (TAP) approach is based on mean-field theory and was first suggested by [1]. There are several ways to derive this approximation [2]. Here we follow the lines of [3, 4] using the *Plefka expansion* [5]. The major difference in our calculation is that $x \in \{0, 1\}$ instead of $\{-1, 1\}$.

The Kullback-Leibler (KL) divergence between two probability mass functions is given by

$$D_{\mathrm{KL}}(q\|p) = \sum_{\mathbf{x}} q(\mathbf{x}) \log \frac{q(\mathbf{x})}{p(\mathbf{x})}, \tag{1}$$

For the exponential family distribution $p(\boldsymbol{x}) = \exp(\boldsymbol{\theta}_p' \mathbf{F}(\mathbf{x}) - \psi_p)$, it is written as [6]

$$D_{\mathrm{KL}}(q\|p) = \phi_q - \boldsymbol{\theta}_p' \boldsymbol{\eta}_q + \psi_p, \tag{2}$$

where ϕ_q is the negative entropy of $q(\mathbf{x})$ and $\boldsymbol{\eta}_q = \langle \mathbf{F}(\mathbf{x}) \rangle_q$. Here our goal is to find $p(\mathbf{x})$ that minimizes the KL divergence given $q(\mathbf{x})$. This is equivalent to maximizing $\boldsymbol{\theta}_p' \boldsymbol{\eta}_q - \psi_p$. If $q(\mathbf{x})$ is an empirical distribution, this is also equivalent to maximizing likelihood of the model. Below we identify $\boldsymbol{\theta}_p$ with the one that maximizes the likelihood given $q(\mathbf{x})$. At this point, the expectation of $\mathbf{F}(\mathbf{x})$ by $p(\mathbf{x})$ is identical to $\boldsymbol{\eta}_q$ ($\langle \mathbf{F}(\mathbf{x}) \rangle_p = \langle \mathbf{F}(\mathbf{x}) \rangle_q$). Hence by dropping the subscripts, the maximized likelihood is written as

$$\phi(\boldsymbol{\eta}) = \boldsymbol{\theta}' \boldsymbol{\eta} - \psi(\boldsymbol{\theta}), \tag{3}$$

which is also the negative entropy of $p(\mathbf{x})$. We also note the relation:

$$\frac{\partial \psi}{\partial \boldsymbol{\theta}} = \boldsymbol{\eta}. \tag{4}$$

Eqs 3 and 4 represent the *Legendre transform*: a translation of a functional relation from $\psi(\boldsymbol{\theta})$ to $\phi(\boldsymbol{\eta})$.

For our model $p(\mathbf{x})$ we now introduce a single scalar α into the distribution which controls the strength of interactions

$$p(\mathbf{x}) = \exp\left(\sum_i \theta_i x_i + \frac{\alpha}{2} \sum_{i \neq j} \theta_{ij} x_i x_j - \psi \right). \tag{5}$$

The model becomes an independent model when $\alpha = 0$. Here the log partition function is a function of $\{\theta_i\}$ and $\{\alpha\theta_{ij}\}$. We now change the variables $\{\theta_i\}$ to $\{\eta_i\}$ by the Legendre transformation of the log partition function to obtain a new free energy:

$$\tilde{\phi}(\{\eta_i\}, \{\alpha\theta_{ij}\}) = \sum_i \theta_i \eta_i - \psi(\{\theta_i\}, \{\alpha\theta_{ij}\}). \tag{6}$$

The function $\tilde{\phi}$ is a function of η_i, θ_{ij}, and α. By assuming weak pairwise interactions because of small α, we approximate $\tilde{\phi}$ by expanding it around the independent model:

$$\tilde{\phi}(\alpha) = \tilde{\phi}|_{\alpha=0} + \left.\frac{\partial\tilde{\phi}}{\partial\alpha}\right|_{\alpha=0} \alpha + \frac{1}{2} \left.\frac{\partial^2\tilde{\phi}}{\partial\alpha^2}\right|_{\alpha=0} \alpha^2 + \cdots \tag{7}$$

The TAP approximation is obtained using expansions up to α^2. By setting $\alpha = 1$, the approximated free energy $\tilde{\phi}$ is obtained as

$$\begin{aligned} \tilde{\phi}(1) \approx & \sum_{i=1}^{N} (\eta_i \log \eta_i + (1-\eta_i)\log(1-\eta_i)) - \frac{1}{2}\sum_{j\neq i} \theta_{ij}\eta_i\eta_j \\ & - \frac{1}{8}\sum_{j\neq i} \theta_{ij}^2 (\eta_i - \eta_i^2)(\eta_j - \eta_j^2). \end{aligned} \tag{8}$$

This approach is called the Plefka expansion method [5]. The first term is the negative entropy of the independent model whereas the second and third terms are obtained by computing derivatives of the negative entropy w.r.t. α. Derivation of the last two terms are given as Eqs 13 and 15 in the end of this section.

By taking the derivative w.r.t. η_i in Eq 8, we obtain a system of self-consistent equations

$$\theta_i = \log\left(\frac{\eta_i}{1-\eta_i}\right) - \sum_{j\neq i}\theta_{ij}\eta_j - \frac{1}{2}\sum_{j\neq i}\theta_{ij}^2\left(\frac{1}{2}-\eta_i\right)\left(\eta_j - \eta_j^2\right). \tag{9}$$

Taking the derivative of Eq 9 w.r.t. η_j, we obtain the (i, j) element of the inverse Fisher information matrix (for θ_is):

$$[\mathbf{G}^{-1}]_{ij} = \frac{1}{\eta_i(1-\eta_i)}\delta_{ij} - \theta_{ij} - \theta_{ij}^2\left(\frac{1}{2}-\eta_i\right)\left(\frac{1}{2}-\eta_j\right). \tag{10}$$

Here δ_{ij} is the Kronecker delta function, where it is 1 if $i = j$ and 0 otherwise. We also let $\theta_{ii} = 0$ for $i = 1, \ldots, N$. Using these formulas we can solve the *forward problem*, i.e., given $\boldsymbol{\theta}$ obtain an approximation for $\boldsymbol{\eta}$. First Eq 9 is solved numerically to get $\{\eta_i\}$. Then we obtain the upper left part of the inverse Fisher information matrix by Eq 10 and invert it. By Eq 16 (main text), we see that η_{ij}s are given by

$$\eta_{ij} = [\mathbf{G}]_{ij} + \eta_i\eta_j. \tag{11}$$

Finally, the inverse Legendre transformation yields the TAP approximation of the log partition function,

$$\begin{aligned} \psi_{\text{TAP}} \approx & \sum_i \theta_i\eta_i - \tilde{\phi}(1) \\ = & \sum_i \theta_i\eta_i - \sum_{i=1}^{N}\{\eta_i\log\eta_i + (1-\eta_i)\log(1-\eta_i)\} + \frac{1}{2}\sum_{j\neq i}\theta_{ij}\eta_i\eta_j \\ & + \frac{1}{8}\sum_{j\neq i}\theta_{ij}^2(\eta_i - \eta_i^2)(\eta_j - \eta_j^2). \end{aligned} \tag{12}$$

Here we use $\{\eta_i\}$ obtained at Eq 9.

Below we compute derivatives of the negative entropy function. Let the hamiltonian of the system be $H = H^{ext} + \alpha H^{int}$, where $H^{ext} = -\sum_i \theta_i x_i$ and $H^{int} = -\frac{1}{2}\sum_{i\neq j}\theta_{ij}x_i x_j$. We reiterate that $\tilde{\phi}$ is a function of mixture coordinates $(\{\eta_i\}, \{\alpha\theta_{ij}\})$ whereas $\{\theta_i\}$ and H^{ext} are dependent on these parameters.

The first derivative is given as

$$\begin{aligned}
\frac{\partial\tilde{\phi}}{\partial\alpha} =& \sum_{i=1}^{N}\frac{\partial\theta_i}{\partial\alpha}\eta_i - \frac{\partial}{\partial\alpha}\log\sum_{\mathbf{x}}\exp(-H) \\
=& \sum_{i=1}^{N}\frac{\partial\theta_i}{\partial\alpha}\eta_i + \frac{1}{\sum_{\mathbf{x}}\exp(-H)}\sum_{\mathbf{x}}\exp(-H)\left[H^{int} + \frac{\partial H^{ext}}{\partial\alpha}\right] \\
=& \sum_{i=1}^{N}\frac{\partial\theta_i}{\partial\alpha}\eta_i + \sum_{\mathbf{x}}\exp(-H-\psi)\left[H^{int} + \frac{\partial H^{ext}}{\partial\alpha}\right] \\
=& \sum_{i=1}^{N}\frac{\partial\theta_i}{\partial\alpha}\eta_i + \left\langle H^{int}\right\rangle_\alpha + \left\langle\frac{\partial H^{ext}}{\partial\alpha}\right\rangle_\alpha \\
=& \left\langle H^{int}\right\rangle_\alpha,
\end{aligned} \tag{13}$$

where $H = H^{ext} + \alpha H^{int}$ and $\langle\cdot\rangle_\alpha$ is the expectation w.r.t. Eq 5 which depends on α. Substituting $\alpha = 0$ yields

$$\left.\frac{\partial\tilde{\phi}}{\partial\alpha}\right|_{\alpha=0} = \left\langle -\frac{1}{2}\sum_{j\neq i}\theta_{ij}x_i x_j\right\rangle_{\alpha=0} = -\frac{1}{2}\sum_{j\neq i}\theta_{ij}\eta_i\eta_j. \tag{14}$$

The second derivative is given as

$$\begin{aligned}
\frac{\partial^2\tilde{\phi}}{\partial\alpha^2} =& \frac{\partial\tilde{\phi}}{\partial\alpha}\left\langle H^{int}\right\rangle_\alpha = \frac{\partial}{\partial\alpha}\sum_{\mathbf{x}}\exp(-H-\psi)H^{int} \\
=& \sum_{\mathbf{x}}\left[\exp(-H-\psi)\left(-\frac{\partial}{\partial\alpha}H - \frac{\partial}{\partial\alpha}\psi\right)H^{int}\right] \\
=& \left\langle\left(-\frac{\partial}{\partial\alpha}H - \left\langle-\frac{\partial H}{\partial\alpha}\right\rangle_\alpha\right)H^{int}\right\rangle_\alpha \\
=& \left\langle\left(-\frac{\partial}{\partial\alpha}H^{ext} - H^{int} - \left\langle-\frac{\partial}{\partial\alpha}H^{ext} - H^{int}\right\rangle_\alpha\right)H^{int}\right\rangle_\alpha \\
=& \left\langle\left(\sum_i\frac{\partial\theta_i}{\partial\alpha}x_i - H^{int} - \left\langle\sum_i\frac{\partial\theta_i}{\partial\alpha}x_i\right\rangle_\alpha + \left\langle H^{int}\right\rangle_\alpha\right)H^{int}\right\rangle_\alpha \\
=& \left\langle\left(\sum_i\frac{\partial\theta_i}{\partial\alpha}(x_i - \eta_i) - H^{int} + \left\langle H^{int}\right\rangle_\alpha\right)H^{int}\right\rangle_\alpha.
\end{aligned} \tag{15}$$

Substituting $\alpha = 0$ yields

$$\begin{aligned}\left.\frac{\partial^2\tilde{\phi}}{\partial\alpha^2}\right|_{\alpha=0} &= \left\langle\left(\sum_i \frac{\partial\theta_i}{\partial\alpha}(x_i-\eta_i) - H^{int} + \left\langle H^{int}\right\rangle_{\alpha=0}\right) H^{int}\right\rangle_{\alpha=0} \\ &= \left\langle\left(-\sum_{j\neq i}\theta_{ij}\eta_j(x_i-\eta_i) + \frac{1}{2}\sum_{j\neq i}\theta_{ij}x_ix_j - \frac{1}{2}\sum_{j\neq i}\theta_{ij}\eta_i\eta_j\right) H^{int}\right\rangle_{\alpha=0} \\ &= -\frac{1}{2}\left\langle\left(-\sum_{j\neq i}\theta_{ij}\eta_jx_i + \frac{1}{2}\sum_{j\neq i}\theta_{ij}\eta_j\eta_i + \frac{1}{2}\sum_{j\neq i}\theta_{ij}x_ix_j\right)\sum_{j\neq i}\theta_{ij}x_ix_j\right\rangle_{\alpha=0} \\ &= -\frac{1}{4}\sum_{j\neq i}\theta_{ij}^2(\eta_i-\eta_i^2)(\eta_j-\eta_j^2). \end{aligned} \quad (16)$$

For the last equality we made use of:

$$\begin{aligned}\left.\frac{\partial\theta_k}{\partial\alpha}\right|_{\alpha=0} = \left.\frac{\partial^2\tilde{\phi}}{\partial\alpha\partial\eta_k}\right|_{\alpha=0} &= \left.\frac{\partial^2\tilde{\phi}}{\partial\eta_k\partial\alpha}\right|_{\alpha=0} \\ &= \frac{\partial}{\partial\eta_k}\left\langle H^{int}\right\rangle_{\alpha=0} = -\frac{1}{2}\frac{\partial}{\partial\eta_k}\sum_{j\neq i}\theta_{ij}\eta_i\eta_j = -\sum_{j\neq k}\theta_{kj}\eta_j. \end{aligned} \quad (17)$$

References

1. Thouless DJ, Anderson PW, Palmer RG. Solution of'solvable model of a spin glass'. Philosophical Magazine. 1977;35(3):593–601.

2. Opper M, Saad D. Advanced mean field methods: Theory and practice. MIT Press; 2001.

3. Tanaka T. Mean-field theory of Boltzmann machine learning. Physical Review E. 1998;58(2):2302.

4. Tanaka T. A theory of mean field approximation. Advances in Neural Information Processing Systems. 1999; p. 351–360.

5. Plefka T. Convergence condition of the TAP equation for the infinite-ranged Ising spin glass model. Journal of Physics A: Mathematical and General. 1982;15(6):1971.

6. Amari Si. Information geometry on hierarchy of probability distributions. Information Theory, IEEE Transactions on. 2001;47(5):1701–1711.
