# Supplementary material for: Approximate Inference for Time-Varying Interactions and Macroscopic Dynamics of Neural Populations
Source: PLoS Comput Biol. 2017 Jan 17;13(1):e1005309. doi: 10.1371/journal.pcbi.1005309 (PMC5283755; doi:10.1371/journal.pcbi.1005309)
Supplement: S3 Text — (PDF) [file pcbi.1005309.s003.pdf]

Approximate Inference for Time-varying Interactions and Macroscopic Dynamics of Neural Populations

Christian Donner[1, 2, 3], Klaus Obermayer[1, 2], Hideaki Shimazaki[4]

1 Bernstein Center for Computational Neuroscience, Berlin, Germany
2 Neural Information Processing Group, Department of Electrical Engineering and Computer Science, Technische Universität Berlin, Berlin, Germany
3 Group for Methods of Artificial Intelligence, Department of Electrical Engineering and Computer Science, Technische Universität Berlin, Berlin, Germany
4 RIKEN Brain Science Institute, Wako-shi, Saitama, Japan

* shimazaki@brain.riken.jp

S3 Text. Generation of simulated data.

Here we explain how the underlying model parameters for Figs 1-4 are generated, and how the artificial spike data is sampled from the model. We discuss the model parameters used to generate the subpopulation activity. Benefit of constructing a large network as combination of independent small subpopulations is that we can exactly compute macroscopic network states (sparsity, entropy, and heat capacity). An additional advantage is that one can exactly sample spiking data without utilizing Monte Carlo methods. Furthermore, this way we do not need to scale the standard deviation of interactions to compare different network sizes.

In order to construct smooth dynamics, the underlying time-varying parameters $\boldsymbol{\theta}_{1:T}$ are sampled as Gaussian processes of $T = 500$ time bins, for $i, j = 1, \ldots, N$:

$$\begin{aligned} \theta_i^{1:T} &\sim \mathcal{GP}(\boldsymbol{\mu}, \mathbf{K}), \\ \theta_{ij}^{1:T} &\sim \mathcal{GP}(\mathbf{0}, \mathbf{K}), \end{aligned} \tag{1}$$

where $\boldsymbol{\mu}$ is a mean vector of size T, and $\mathbf{K}$ is the $T \times T$ covariance matrix. For $\theta_i^{1:T}$, the mean vector $\boldsymbol{\mu} = (\mu_1, \ldots, \mu_T)$ is modulated using an inverse Gaussian function as

$$\mu_t = \begin{cases} -2 \text{ for } t < 100 \\ -2 + \frac{\lambda}{2\pi g(t)^3} \exp\left(-\frac{\lambda}{2f(t)}(g(t)-1)^2\right) \text{ for } t \geq 100, \end{cases} \tag{2}$$

where $g(t) = 3(t - 100)/400$ and $\lambda = 3$. For $\theta_{ij}^{1:T}$, the mean is fixed at zero. To produce smooth processes, the covariance matrix $\mathbf{K}$ dictating the smoothness for both θ_i and θ_{ij} is chosen as

$$[\mathbf{K}]_{t,t'} = \frac{1}{\sigma_1} \exp\left(-\frac{|t - t'|^2}{2\sigma_2^2}\right), \tag{3}$$

where $\sigma_1 = 12$ and $\sigma_2 = 50$. While the processes of the first order natural parameters $\{\theta_i^{1:T}\}$ have time-varying mean at different time points, it should be noted that the sampled interactions $\{\theta_{ij}^{1:T}\}$ also smoothly change over time.
