# Supplementary material for: Approximate Inference for Time-Varying Interactions and Macroscopic Dynamics of Neural Populations
Source: PLoS Comput Biol. 2017 Jan 17;13(1):e1005309. doi: 10.1371/journal.pcbi.1005309 (PMC5283755; doi:10.1371/journal.pcbi.1005309)
Supplement: S4 Text — (PDF) [file pcbi.1005309.s004.pdf]

Approximate Inference for Time-varying Interactions and Macroscopic Dynamics of Neural Populations

Christian Donner[1, 2, 3], Klaus Obermayer[1, 2], Hideaki Shimazaki[4]

1 Bernstein Center for Computational Neuroscience, Berlin, Germany
2 Neural Information Processing Group, Department of Electrical Engineering and Computer Science, Technische Universität Berlin, Berlin, Germany
3 Group for Methods of Artificial Intelligence, Department of Electrical Engineering and Computer Science, Technische Universität Berlin, Berlin, Germany
4 RIKEN Brain Science Institute, Wako-shi, Saitama, Japan

* shimazaki@brain.riken.jp

S4 Text. Simulated experiment with a balanced network.

To examine consequences of the proposed statistical analysis on a physiologically plausible model of cortical networks, we used a well-studied balanced network model (see Fig 3 of [1]) with slight modifications. For the network simulations, we use the Brian simulator [2]. The network consists out of 3 distinct populations: Input ($N = 800$), excitatory ($N = 800$) and inhibitory ($N = 200$) neurons. Connectivity and parameters of the conductance-based leaky integrate-and-fire neurons are set as in the original work. In contrast to the cited paper the inputs provided to the network are inhomogeneous Poisson processes whose firing rates are all given by

$$r(t) = 7.5 \text{ Hz} + 5 \cdot stim(t) \exp\left(-(\nu_{stim} - \nu_{pref})^2\right) \text{ Hz}, \tag{1}$$

where $stim(t) = 1$ if a stimulus is present, and 0 otherwise. $\nu_{stim} \in [-\pi, \pi)$ is the orientation of the stimulus. The preferred direction of each input is drawn from a uniform distribution $\nu_{pref} \sim [-\pi, \pi)$.

The following experiment was simulated 1000 times with this network. A simulation started with activity without any stimulation for 1 s. Then a stimulus with $\nu_{stim} = \pi$ is shown to the network for 2 s. A period of 0.5 s in the absence of stimulus follows.

During the experiment the spike times of 100 randomly selected excitatory and 40 inhibitory neurons are recorded. For the statistical analysis the 40 excitatory and the 20 inhibitory neurons are chosen that exhibit the highest firing rates in the recorded population.

References

1. Renart A, de la Rocha J, Bartho P, Hollender L, Parga N, Reyes A, et al. The asynchronous state in cortical circuits. Science. 2010;327(5965):587–590.
2. Stimberg M, Goodman DF, Benichoux V, Brette R. Equation-oriented specification of neural models for simulations. Frontiers in Neuroinformatics. 2014;8:6.
